# Prodomain Removal Enables Neto to Stabilize Glutamate Receptors at the *Drosophila* Neuromuscular Junction

**DOI:** 10.1371/journal.pgen.1004988

**Published:** 2015-02-27

**Authors:** Young-Jun Kim, Oghomwen Igiesuorobo, Cathy I. Ramos, Hong Bao, Bing Zhang, Mihaela Serpe

**Affiliations:** 1 Program in Cellular Regulation and Metabolism, NICHD, NIH, Bethesda, Maryland, United States of America; 2 Division of Biological Sciences, University of Missouri, Columbia, Missouri, United States of America; The University of North Carolina at Chapel Hill, United States of America

## Abstract

Stabilization of neurotransmitter receptors at postsynaptic specializations is a key step in the assembly of functional synapses. *Drosophila* Neto (Neuropillin and Tolloid-like protein) is an essential auxiliary subunit of ionotropic glutamate receptor (iGluR) complexes required for the iGluRs clustering at the neuromuscular junction (NMJ). Here we show that optimal levels of Neto are crucial for stabilization of iGluRs at synaptic sites and proper NMJ development. Genetic manipulations of Neto levels shifted iGluRs distribution to extrajunctional locations. Perturbations in Neto levels also produced small NMJs with reduced synaptic transmission, but only Neto-depleted NMJs showed diminished postsynaptic components. *Drosophila* Neto contains an inhibitory prodomain that is processed by Furin1-mediated limited proteolysis. *neto* null mutants rescued with a Neto variant that cannot be processed have severely impaired NMJs and reduced iGluRs synaptic clusters. Unprocessed Neto retains the ability to engage iGluRs in vivo and to form complexes with normal synaptic transmission. However, Neto prodomain must be removed to enable iGluRs synaptic stabilization and proper postsynaptic differentiation.

## Author Summary

Synapse development is initiated by genetic programs, but is coordinated by neuronal activity, by communication between the pre- and postsynaptic compartments, and by cellular signals that integrate the status of the whole organisms and its developmental progression. The molecular mechanisms underlining these processes are poorly understood. In particular, how neurotransmitter receptors are recruited and stabilized at central synapses remain the subject of intense research. The *Drosophila* NMJ is a glutamatergic synapse similar in composition and physiology with mammalian central excitatory synapses. Like mammals, *Drosophila* utilizes auxiliary subunit(s) to modulate the formation and function of glutamatergic synapses. We have previously reported that Neto is an auxiliary protein essential for functional glutamate receptors and for organization of postsynaptic specializations. Here we report that synapse assembly and NMJ development are exquisitely sensitive to postsynaptic Neto levels. Furthermore, we show that Neto activity is controlled by Furin-type proteases, which regulate the processing and maturation of many developmentally important proteins, from growth factors and neuropeptides to extracellular matrix components. Such concerted control may serve to coordinate synapse assembly with synapse growth and developmental progression.

## Introduction

Synapse development is a highly orchestrated process that enables proper establishment of neural circuits and development of the nervous system. Crucial to synapse assembly is the recruitment and stabilization of neurotransmitter receptor complexes at synaptic sites [1]. Receptor complexes can be inserted directly into synaptic membranes via vesicular trafficking from ER-Golgi network, or they can move into the synaptic regions by lateral diffusion from extrasynaptic pools (reviewed in [2,3]). Clustering of neurotransmitter receptors at new synapses induces expression of synaptic components and assembly of postsynaptic structures, such as postsynaptic densities (PSDs), which in turn help maintain the local density of receptors [4]. Neural activity and trans-synaptic communication between pre- and postsynaptic specializations together with intracellular signals within the synaptic partners themselves ensure the maturation, refinement and plasticity of the synaptic connections and synapse growth [5–9]. The molecular mechanisms that coordinate the recruitment and stabilization of receptors at synaptic sites and assembly of synaptic structures with synaptic growth remain unclear.

The *Drosophila* NMJ provides an ideal genetic system to examine the mechanisms that couple synapse assembly with synapse growth and development. The fly NMJ is a glutamatergic synapse similar in composition and physiology to vertebrate AMPA/kainate central synapses [10,11]. The fly NMJ iGluRs are tetrameric complexes composed of three essential subunits, GluRIIC, GluRIID and GluRIIE, absolutely required for assembling functional channels [12–14]. The fourth subunit can be either GluRIIA (type-A channels) or GluRIIB (type-B) [15–17]. GluRIIA and GluRIIB compete for the essential subunits, which are limiting for the formation of functional receptors. Before a muscle is innervated, low levels of iGluRs are present diffusely in the muscle membrane. Innervation triggers the clustering of iGluRs at synaptic locations and postsynaptic differentiation [18–20]. Type-A channels are the first to arrive at nascent synapses, while type-B, which desensitize ten times faster than type-A, mark more mature synapses [12,20,21]. The fly NMJ iGluRs, but not other PSD components, show very little turnover suggesting that the iGluR complexes are stably incorporated at synaptic sites [22].

At the *Drosophila* NMJ, clustering of iGluRs and formation of postsynaptic specializations requires an additional essential protein, Neto [23]. Neto belongs to a family of highly conserved transmembrane proteins sharing an ancestral role in the formation and modulation of glutamatergic synapses [24–26]. Vertebrate Neto proteins (Neto1 and 2) and *C*. *elegans* Neto/Sol-2 have emerged as auxiliary subunits that modulate the gating properties of AMPA/kainate-type channels and their synaptic localization without influencing their delivery to the cell surface [24–29]. Likewise, *Drosophila* Neto associates with iGluRs in vivo and controls their trafficking and clustering at NMJ synapses without affecting their muscle expression levels [23]. Reduced synaptic iGluRs alter the function of NMJs causing locomotor defects and reduced synaptic transmission [12,30]. Lack of junctional iGluRs also induces a cascade of defects in the assembly and maintenance of postsynaptic specializations [23,30]. For example, Neto- or iGluRs-deprived synapses have reduced accumulation of PSD components, such as p21-activated kinase (PAK), and sparse subsynaptic reticulum (SSR), a structure comprised of stacks of muscle membranes surrounding and stabilizing synaptic boutons [31].

Intriguingly, synapses developing at suboptimal Neto/iGluR levels share a number of morphological and physiological defects with mutants in the BMP signaling, a pathway that controls the NMJ growth and confers synaptic homeostasis [32]. Similar to *neto* mutants, BMP mutant NMJs have fewer boutons and reduced excitatory junctional potential (EJP) (reviewed in [32]). Furthermore, Neto in complex with type-A receptors promote the phosphorylation and accumulation of the BMP pathway effector Mad at synaptic locations [33]. The BMP-type signaling factors are produced as inactive precursors, with inhibitory prodomains that must be removed by proprotein convertases to generate the active ligands [34]. Furin-type proteases control the limited proteolysis of inactive BMP precursors and directly regulate their activities [35–37]. In many tissues, sequential processing of BMP prodomains modulates the range and signaling activities of BMP ligands [38]. At the *Drosophila* NMJ additional TGF-β factors regulate the expression of Glass bottom boat (Gbb), a BMP7 homolog required for the BMP retrograde signaling [39,40]. Furin-type proteases activate all these TGF-β-type factors as well as the BMP-1/Tolloid enzymes that augment TGF-β signaling indicating that Furins provide an important means for controlling cellular signaling at the *Drosophila* NMJ.

Here we report that Neto protein levels are critical for synaptic trafficking and clustering of iGluRs. Excess or reduced Neto protein in the striated muscle induced formation of NMJs with reduced number of synaptic boutons, decreased synaptic iGluRs and diminished neurotransmission. Neto activities are regulated by Furin-mediated proteolysis and removal of an inhibitory prodomain. In the absence of prodomain cleavage, Neto engages the iGluRs but fails to promote their recruitment and stable incorporation at synaptic sites and to initiate postsynaptic differentiation. Since Furins also cleave and activate signaling molecules, such as TGF-β factors, Furins may synchronize the processing of Neto and TGF-β to control synaptic growth.

## Results

### Optimal expression level is required for Neto functions

**Fig 1:**
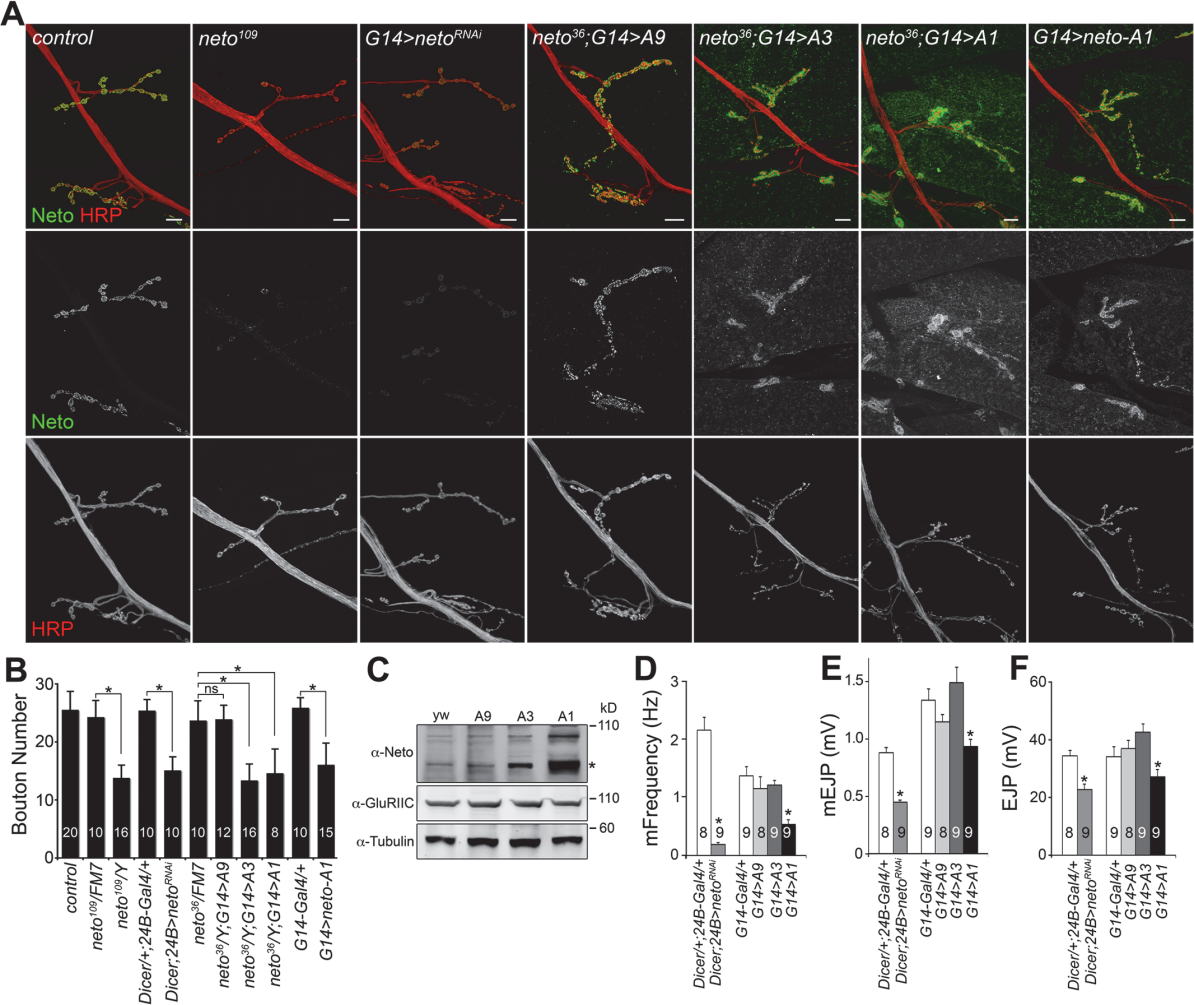
Neto levels affect NMJ development and function. (A) Confocal images of NMJ4 (segment A4) in third instar larvae of indicated genotypes labeled for Neto (green) and HRP (red). Excess Neto distributed in larger, more diffuse synaptic clusters or spread over the muscle. Bouton numbers were decreased at low Neto levels in the *neto^109^* hypomorph or in RNAi knockdown (*24B>neto^RNAi^*) (quantified in B). Excess Neto also induced smaller NMJs in a concentration-dependent manner in both rescue (*neto^36^/Y;G14>neto-Ai*) or overexpression (*G14>neto-A1*) settings. (C) Western blot comparison of Neto, GluRIIC and Tubulin levels in muscle extracts from *neto* null larvae rescued with various *neto* transgenes. Neto levels vary from low (*A9*) and moderate (*A3*) to high (*A1*). (*) processed Neto. (D-F) Electrophysiological recordings of control and *neto* loss-of-function and gain-of-function third instar larvae. The mEJPs frequency (D) and amplitude (E) as well as EJP amplitude (F) were reduced at NMJs with reduced or high levels of Neto. The numbers of NMJs examined are indicated in each bar. Error bars indicate SEM. *; *p*<0.001, ***; *p*<0.05. Bars: 10 μm.

Similar to *neto* hypomorphs, RNAi-mediated knockdown of Neto in the striated muscle altered NMJ development (Fig. 1A, B and [23,33]). Interestingly, *neto* overexpression in the muscle also induced abnormal synapse development. We rescued *neto* null mutants (*neto* ^*36*^) with *neto* transgenes with various expression levels and found that excess Neto accumulated at NMJ synapses and extrajunctional locations in a dose-dependent manner (Fig. 1A, C). Low to moderate levels of Neto clustered at synaptic sites (i.e. using *neto-A9* transgene), but excess Neto (*neto-A3*, or *neto-A1* for the highest level) had predominantly diffuse distribution with fewer individual synaptic puncta and abundant extrasynaptic signals. Similar patterns were found in animals with overexpressed *neto* transgene (where *neto-A1* induced the strongest phenotypes). Excess Neto had detrimental effects on the viability of rescued animals at all stages of development (S1 Fig.). Neto levels also affected NMJ growth. In larvae with either reduced or excess Neto, the number of boutons was decreased although the branching patterns differed: longer branches at reduced Neto and shorter branches at excess Neto (Figs 1A, S1). This suggests that independent signaling pathways control NMJ growth and bouton formation.

To examine the effects of Neto levels on synapse function we recorded excitatory junction potentials (EJPs) and spontaneous miniature potentials (mEJPs, or minis) from muscle 6 of third instar larvae (Fig. 1D-F). In control larvae (*Dicer/+*: *24B-Gal4/+*) minis occurred two times per second on average. This was reduced to 0.3 events per second at Neto-depleted synapses (*dicer; 24B>neto* ^*RNAi*^) similar to that observed in *neto* hypomorphs [23]. Excess Neto showed a reduction in mini frequency, and to a lesser extent in mini amplitude, but only when Neto was expressed at very high levels; larvae with moderate levels of additional Neto had normal mEJPs. The mini frequency appeared particularly sensitive to Neto levels and was significantly reduced in both Neto-depleted and Neto-excess conditions. The reduction in mini frequency and amplitude occurred in muscles with no change in both resting potential and input resistance. The EJP amplitude was similarly sensitive to Neto levels: mild/moderate increase in Neto levels showed no significant change in EJP amplitude, while strong perturbations of the Neto levels (depletion or excess) induced significant reduction in the EJP amplitude.

### Excess Neto reduces synaptic iGluRs

**Fig 2:**
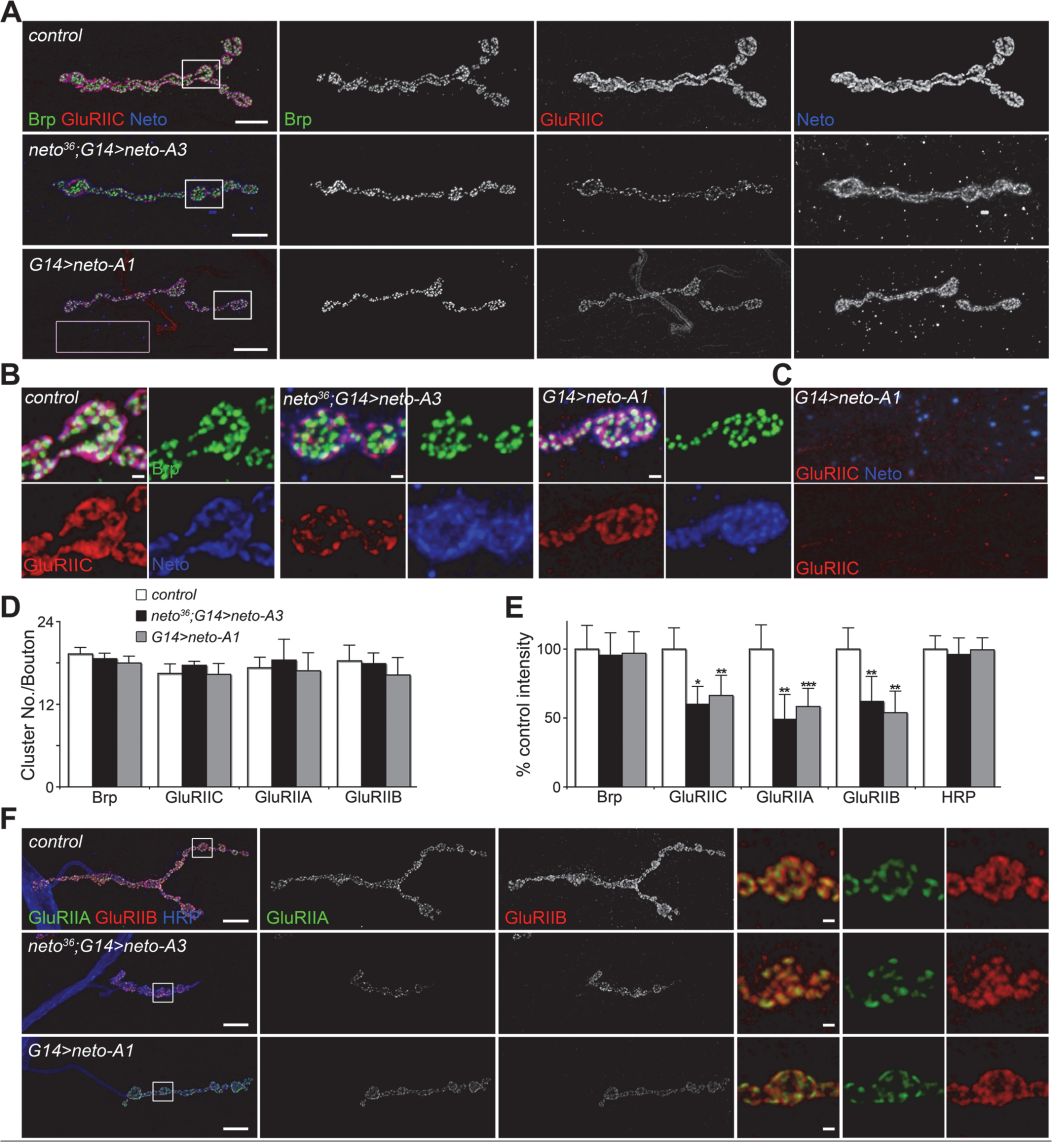
Excess Neto causes reduced iGluR synaptic clusters. (A-B) Confocal images of NMJ4 (A) and bouton details (B) in larvae of indicated genotypes labeled for Brp (green), GluRIIC (red), and Neto (blue). GluRIIC and Neto extrajunctional staining (pink box in A) are shown in (C). While Brp synaptic signals were not affected, GluRIIC showed decreased synaptic localization at excess Neto levels (*neto^36^/Y;G14>neto-A3* or *G14>neto-A1*) (quantified in D-E). Ten NMJs were analyzed and quantified for each genotype. (F) Both type-A and type-B receptors were decreased at NMJs with excess Neto. Images were enhanced in the bouton details (right) for relative IIA/IIB comparisons. Error bars indicate SEM. *; *p*<0.001, **; *p*<0.01. Bars: 10 μm, 1 μm in details.

Although GluRIIC muscle levels were constant in larvae with increased Neto expression (Fig. 1B), the similarities between the NMJ physiological properties at reduced or excess Neto suggest that excess Neto could affect the number and density of postsynaptic iGluRs. Indeed, excess Neto produced a significant decrease of GluRIIC synaptic clusters: the number of synaptic contacts per bouton did not change, but the intensity of the GluRIIC synaptic signals was reduced to 58% ± 12% of the control (Fig. 2A-E). The anti-GluRIIC also labeled extrasynaptic puncta that occasionally accompanied small Neto clusters, but did not co-localize with the large extrajunctional Neto-positive puncta, presumably associated with secretory vesicles (Fig. 2C). In contrast, the synaptic distribution of Bruchpilot (Brp), an active zone scaffold [41], remained unaffected by excess Neto, indicating that Neto specifically regulates the distribution of postsynaptic receptors. The decrease of synaptic iGluRs showed no subtype specificities when Neto was overexpressed in a wild-type background (*G14>neto-A1*); both GluRIIA and GluRIIB synaptic levels were similarly decreased (to 60% and respectively 54% from control) (Fig. 2E-F). This is consistent with the normal quantal size (or mini amplitude), observed at these NMJs (Fig. 1E) [15,16]. However, when excess Neto was introduced in the *neto* null background (*neto^36^; G14>neto-A3*), the GluRIIA synaptic levels were reduced slightly more than the GluRIIB, to 48% and respectively 62% from control.

**Fig 3:**
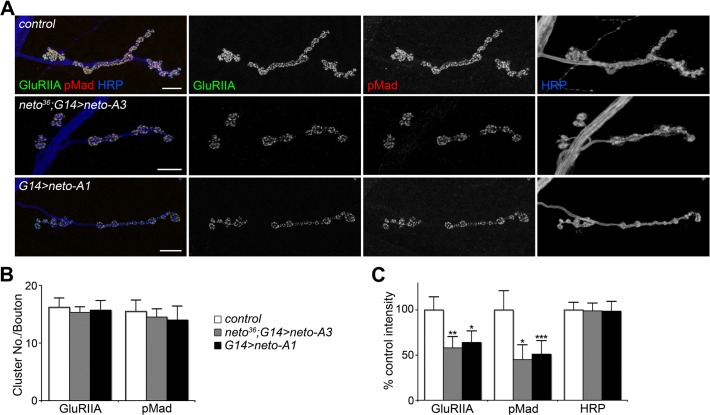
High Neto levels affect the BMP signaling. (A) Confocal images of NMJ4 (segment A4) in larvae of indicated genotypes labeled for GluRIIA (green), pMad (red), and HRP (blue). Excess Neto induced a decrease in the intensity but not number of synaptic GluRIIA and pMad signals (quantified in B-C). All samples were processed and imaged similarly using at least ten NMJs per genotype. Error bars indicate SEM. *; *p*<0.001, **; *p*<0.01, ***; *p*<0.05. Bars: 10 μm.

Loss of synaptic pMad, the BMP pathway effector, correlated with small NMJs with reduced synaptic release in *neto* and *importin-β11* mutants [33,42]. We found that Neto overexpression also caused attenuation of the synaptic pMad levels likely by decreasing the levels of synaptic type-A receptors (Fig. 3). Reduced synaptic iGluRs together with diminished retrograde BMP signaling could explain the small size of NMJs with excess or reduced Neto levels. However, there were several differences between these NMJs. Unlike *neto* hypomorph larvae, which showed diminished synaptic localization of multiple synaptic components, such as p21-activated kinase (PAK), Discs large (Dlg), and α-Spectrin [23], excess Neto did not affect the synaptic accumulation of any of these proteins (S2 Fig.). In line with normal Brp, excess Neto did not affect the presynaptic localization of cysteine-string protein (CSP) [43]. Thus, the *neto* gain-of-function NMJ phenotypes cannot result from insufficient trafficking and recruitment of postsynaptic components. Normal recruitment of Dlg at synaptic locations was also observed when V5- or GFP-tagged Neto variants replaced the endogenous Neto protein (S3 Fig.). Similar to untagged Neto, excess Neto-V5 or Neto-GFP induced smaller NMJs with normal synaptic transmission (Neto-GFP-rescued NMJ shown in S3 Fig.), indicating that the addition of tags did not affect Neto activities and gain-of-function phenotypes (Figs 1, S3 and [23]).

### Neto controls recruitment and stabilization of iGluRs at PSDs

**Fig 4:**
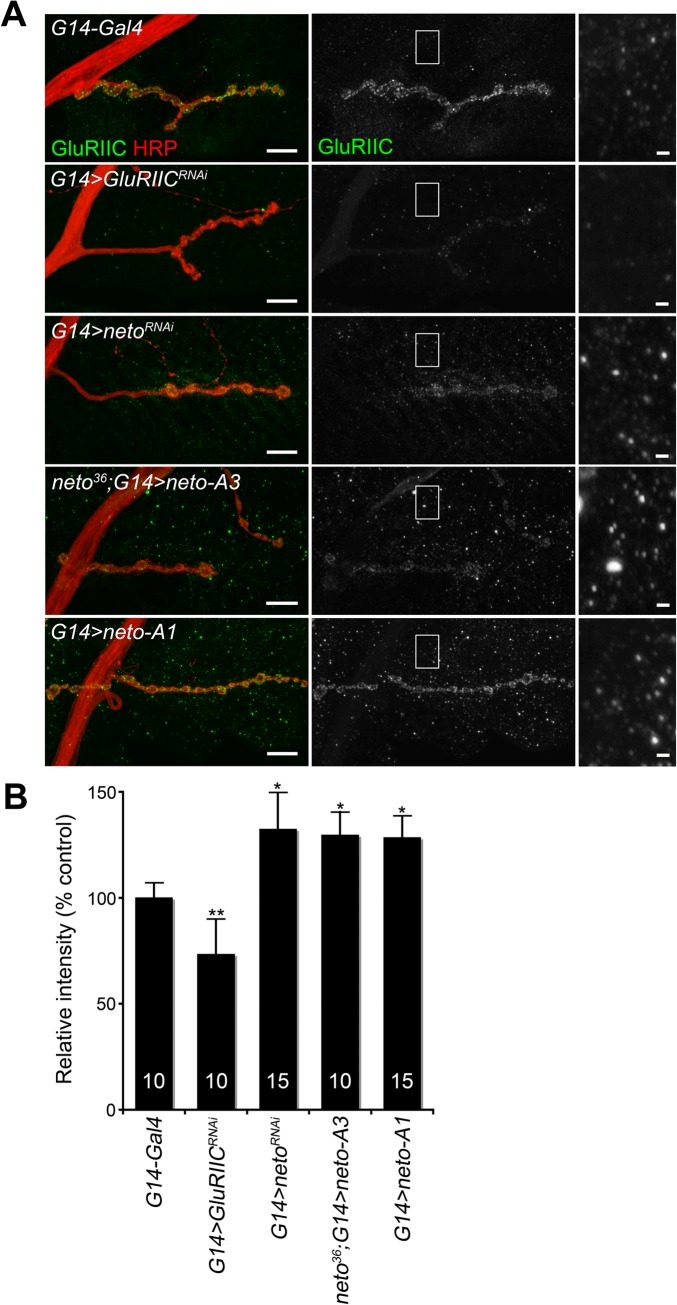
Surface distribution of iGluR complexes at various Neto levels. (A) Confocal images of NMJ4 in third instar larvae of indicated genotypes labeled for GluRIIC (green), and HRP (red). Surface distribution of GluRIIC at synaptic and extrasynaptic locations was captured by immunohistochemistry with an N-terminal-specific antibody in detergent-free conditions. The GluRIIC signals were absent in the knockdown control (*G14>GluRIIC^RNAi^*) and were shifted from synaptic to extrasynaptic locations at either low (*G14>neto^RNAi^*) and excess Neto levels (*neto^36^;G14>neto-A3* and *G14>neto-A1*) (quantified in B). The image insets are shown at higher intensity. The numbers of NMJs examined are indicated in each bar. Error bars indicate SEM. *; *p*<0.001, **; p<0.01. Bars: 10 μm, 1 μm in details.

How could excess Neto diminish the synaptic iGluR levels without affecting any other synaptic components tested here? Stable synaptic receptors are thought to be part of large aggregates organized by proteins secreted from the presynaptic compartment [44,45] and further stabilized by postsynaptic scaffolds [46]. Neto may interact with neuron-secreted proteins that trigger iGluRs synaptic clustering and/or with intracellular motors and scaffolds that promote iGluRs trafficking and stabilization at synaptic sites. Excess Neto may engage in unproductive interactions and overwhelm the cellular machineries involved in the trafficking and clustering of iGluRs at synaptic locations. Since Neto does not affect the net levels of receptor subunits in the postsynaptic muscle (Fig. 1B and [23]), then iGluRs are predicted to accumulate at extrajunctional locations at suboptimal Neto levels. Indeed, genetic manipulation of Neto levels triggered a redistribution of iGluR-positive signals from junctional to extrajunctional locations (Fig. 2C and [23]). Moreover, Neto proteins appear to have no roles in the surface delivery of the iGluRs in vertebrate and in *C*. *elegans* [25,26] suggesting that reduced or excess Neto levels should induce accumulation of extrajunctional iGluRs at the muscle surface. We tested this prediction by staining the larval fillets in detergent-free protocols with antibodies raised against the extracellular domain of GluRIIC. Under these conditions, extrajunctional GluRIIC staining was barely visible in control, but was very prominent on the muscle of larvae with reduced or excess Neto (Fig. 4A-B). Surface accumulation at extrajunctional locations of GluRIIA was also observed in *neto* ^*109*^ hypomorphs [23]. Similar results were obtained in both rescue and overexpression experiments with either *neto-A3* or *neto-A1* transgenes even though *neto-A1* appears to induce a higher Neto expression level (Figs 4, 1). Together, our data indicate that optimal Neto levels are crucial for the recruitment and stabilization of iGluRs at synaptic sites. Similar to vertebrate or *C*. *elegans*, perturbations of the *Drosophila* Neto levels do not appear to affect the surface delivery of iGluRs and instead influence the iGluRs distribution between synaptic and extrasynaptic locations.

### Neto prodomain is cleaved at dibasic residues

**Fig 5:**
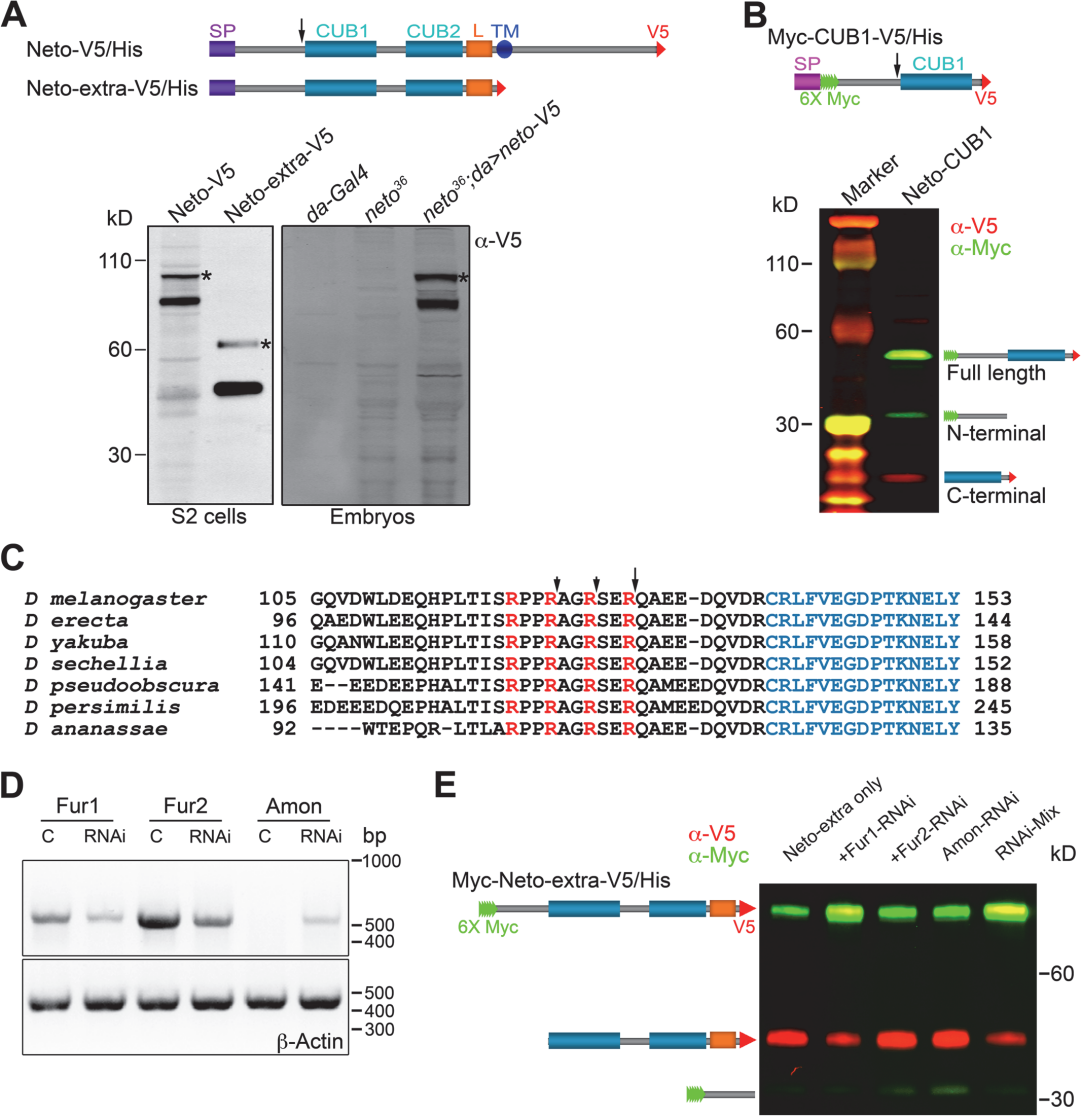
Neto contains a prodomain cleaved by Furin1-mediated limited proteolysis. (A-B) Diagrams and Western blot analyses of various Neto constructs. (A) Neto-V5/His is full-length Neto (SP- signal peptide, CUB1 and -2- the CUB domains, L- LDLa motif, TM- transmembrane domain) C-tagged with V5-6xHis epitopes (V5). Neto-extra-V5 contains only the extracellular part. The majority of tagged Netos were detected as cleaved forms in tissue culture or rescued embryos (*neto^36^;da>neto-V5*). (*) uncleaved proteins. (B) A short variant including the CUB1 domain retained processing; the C-end fragment was used to identify the cleavage site(s). (C) *Drosophila* Neto proteins contain highly conserved Furin consensus sites (arrows). The R-Q bond, 9 residues upstream the CUB1 domain (in blue) appears to be the primary cleavage site. (D-E) RNAi interference in S2 cells showed that Fur1 cleaves Neto. The efficiency of RNAi treatment was verified by RT-PCR and DNA analyses relative to β-Actin control (D). Fur2 did not influence Neto processing. In S2 cells, Amon expression levels were too low to rule out its contribution. The data shown are representative images from five experimental repeats.

Unlike vertebrate or *C*. *elegans* Neto, *Drosophila* Neto contains a long sequence preceding the first CUB domain (CUB1). Full-length Neto is predicted to be a 78 kD protein, yet when expressed in S2 insect cell, Neto runs as two bands: a minor band with relative mobility ~100 kD, and a major band of ~85 kD (Fig. 5A). Truncated Neto variants containing only the extracellular part (Neto-extra) showed bands of ~60 and ~45 kD. Similar pattern was also detected in *neto* ^*36*^ null embryos rescued with a *neto-V5* transgene.

To examine whether Neto is cleaved, we generated a binary-tagged CUB1 fragment (Myc-CUB1-V5/His, Fig. 5B). This secreted fragment produced three distinct bands corresponding to full length, N-terminal and C-terminal fragments. The C-terminal fragment was purified and analyzed by Edman degradation and mass spectrometry. Three cleavage sites within a region containing tandem repeats of RXXR dibasic motifs, upstream of the CUB1 domain, were identified. The major cleavage site appears to be the R^129^-Q bond, but R^126^-S and R^123^-A bonds could also be cleaved (Fig. 5C). Interestingly, this region is highly conserved in all *Drosophila* species but not in vertebrate or *C*. *elegans* Neto, suggesting that this processing has functional implications for Neto functions in flies. The cleavage sites match the consensus processing sequence for Furin-like proprotein convertases (PC), also known as PACE (Paired basic Amino acid Cleaving Enzyme), which process latent precursor proteins into their biologically active forms [47].

*Drosophila* genome codes for three Furin-type enzymes: Furin1 (Fur1), Furin2 (Fur2), and Amontillado (Amon). Fur1 and Fur2 were expressed and analyzed in vitro, but their mutants have not been described yet [48,49]. Mutants in *amon*, encoding the *Drosophila* homolog of the neuropeptide precursor processing protease PC2, display partial embryonic lethality, defective larval growth, and arrest during the first to second instar larval molt [50,51]. To confirm that Furins are responsible for cleaving Neto we used an RNAi approach [36]. We generated double strand RNA (dsRNA) for each of the three Furin-like coding genes, co-transfected them with Neto expression constructs in S2 cells, and examined the protein products. The efficiency of RNAi treatments was verified by RT-PCR (Fig. 5D). We found that knockdown of Fur1 activities reduced the production of the small, cleaved bands and increased the level of unprocessed form (Fig. 5E, lanes 1 and 2). However, we did not find any difference by knocking down Fur2 or Amon (Fig. 5E, lanes 3 and 4). Combination of all 3 different dsRNAs did not further reduce the proportion of uncleaved Neto forms compared to Fur1 RNAi (Fig. 5E, lanes 1 and 5), indicating that Fur1 is the primary enzyme for cleaving *Drosophila* Neto in S2 cells.

In flies, *fur1* is expressed throughout development in multiple tissues including larval central nervous system and carcass [52]. RNAi-mediated *fur1* knockdown in the striated muscle produced NMJs with fewer and smaller boutons, normal Brp synaptic clusters, but significantly diminished levels of synaptic iGluRs (S4 Fig.). While these phenotypes are reminiscent of NMJs with suboptimal Neto, they cannot be solely attributed to reduced Neto activities due to lack of processing. Fur1, like all Furin-type proteases, cleaves and activates multiple developmentally important substrates, including extracellular matrix components and signaling molecules such as TGFβ-type ligands [53]. In fact, stronger RNAi treatments (in the presence of Dicer or at higher rearing temperature) distorted the muscle fibers and induced early larval lethality. A pulse of high temperature (one day at 30°C) also disrupted the muscle structures. Fur1 knockdown also induced significant reduction in GluRIIA and pMad synaptic signals, likely because inefficient activation of precursor TGFβ-type factors, including Gbb (S4 Fig.). Interestingly, down-regulation of *fur1* in motor neurons elicited similar NMJ phenotypes, underscoring the complexity of Fur1-dependent activities.

### Neto prodomain limits stabilization of iGluRs at PSDs

**Fig 6:**
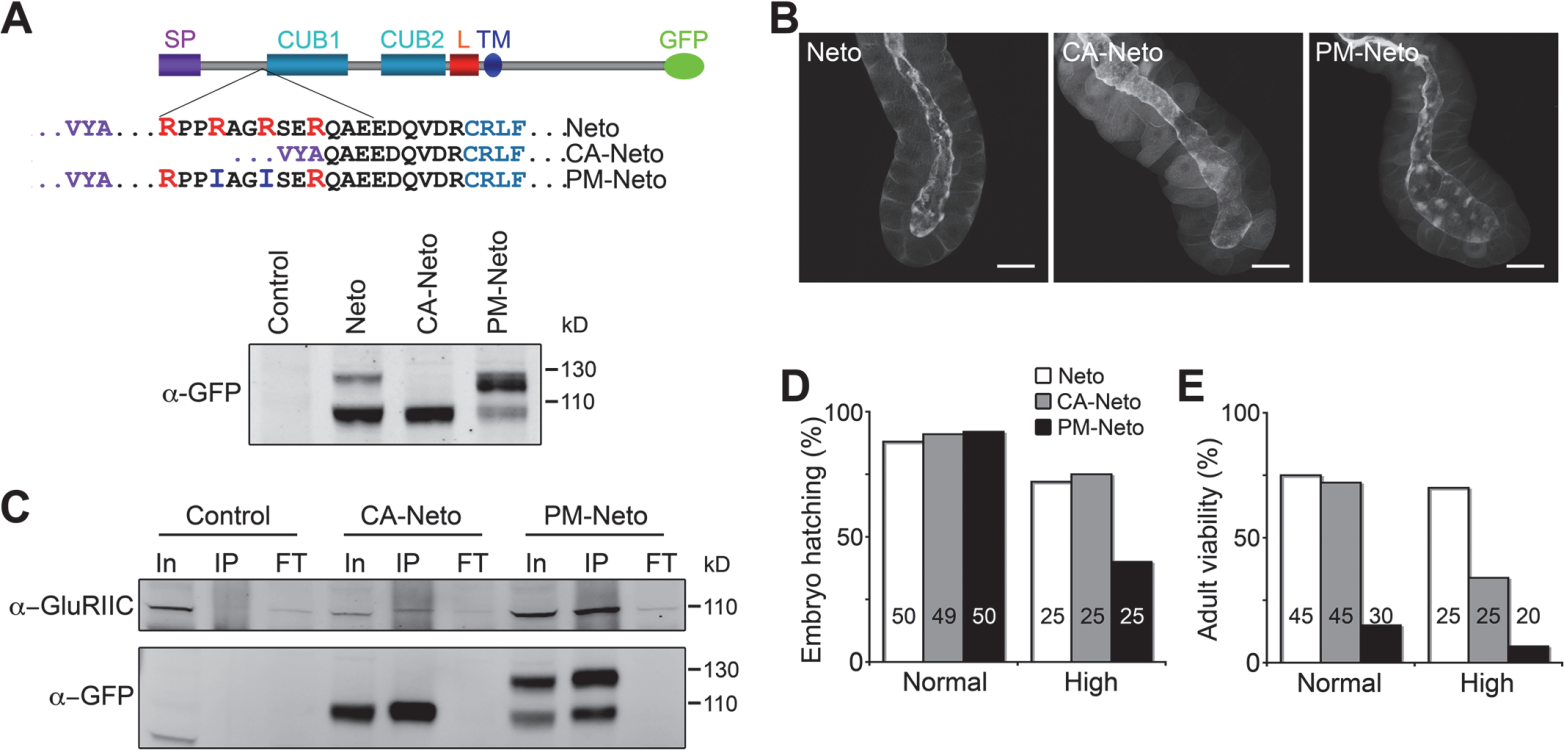
The prodomain restricts Neto activities. (A) Diagram and Western blot analysis of constitutively active (CA) and processing mutant (PM) Neto-GFP variants. (B) Fluorescence images of salivary glands from third instar larvae expressing Neto-GFP variants as indicated (*G14>neto-GFP*). Prodomain processing did not affect the apical localization of Neto. (C) Co-immunoprecipitation (IP: α-GFP, WB: α-GluRIIC) from muscle extracts from control (*y^1^w^1118^*) and *neto* rescued larvae (*neto^36^;G14>CA-neto-GFP* and *neto^36^;G14>PM-neto-GFP*) showed that both CA- and PM-Neto bind iGluRs. (D-E) PM-Neto is less efficient in rescuing the embryonic lethality and adult viability of *neto* null mutants compared with control or CA-Neto. Genotypes: control (*neto^36^;G14>neto-GFP* at 25°C); CA-Neto normal (*neto^36^;G14>CA-neto-GFP-N4* at 18°C); CA-Neto high (*neto^36^;G14>CA-neto-GFP-N4* at 25°C); PM-Neto normal (*neto^36^;G14>PM-neto-GFP-D2* at 25°C); PM-Neto high (*neto^36^;G14>PM-neto-GFP-D1* at 25°C). The numbers of animals analyzed are indicated in each bar. Bars: 10 μm.

To study the biological relevance of Neto processing by Furin-type proteases we generated a constitutively active Neto variant (CA-Neto), without the prodomain, and a processing mutant Neto (PM-Neto), with an uncleavable prodomain (Fig. 6A). When expressed in S2 cells, Neto-GFP was detected as double bands of expected sizes, mostly processed form. CA-Neto-GFP was found as a single, processed protein, while PM-Neto-GFP was predominantly unprocessed. We noticed that a small fraction of PM-Neto (<15%) was processed presumably by promiscuous proteolysis, which may partly remove the prodomain; however, such cleavage usually occurs at ectopic locations, adding or removing additional residues from the processed product. Further mutations in this conserved region did not completely abolish Neto processing, but could impact the proper function of the adjacent CUB domain.

To examine the subcellular distribution of Neto variants we took advantage of the apical localization of Neto in epithelial tissues. *G14-Gal4* drives the expression of *UAS* transgenes in muscles but also in salivary glands. We found that all Neto variants localized to the luminal side of the salivary gland (apical surface), indicating that prodomain processing does not affect membrane targeting and apical localization of Neto proteins (Fig. 6B). Nor did prodomain processing impact the ability of Neto variants to form complexes with iGluRs in the striated muscle. Similar to Neto, CA-Neto and PM-Neto retained the capacity to pull-down iGluRs from muscle extracts (Fig. 6C). However, PM-Neto was severely impaired in its ability to rescue the *neto* null mutants, while CA-Neto generally resembled the Neto control (Fig. 6D, E). Very few PM-Neto rescued animals reached the adult stages: these flies did not fly and had locomotor defects.

**Fig 7:**
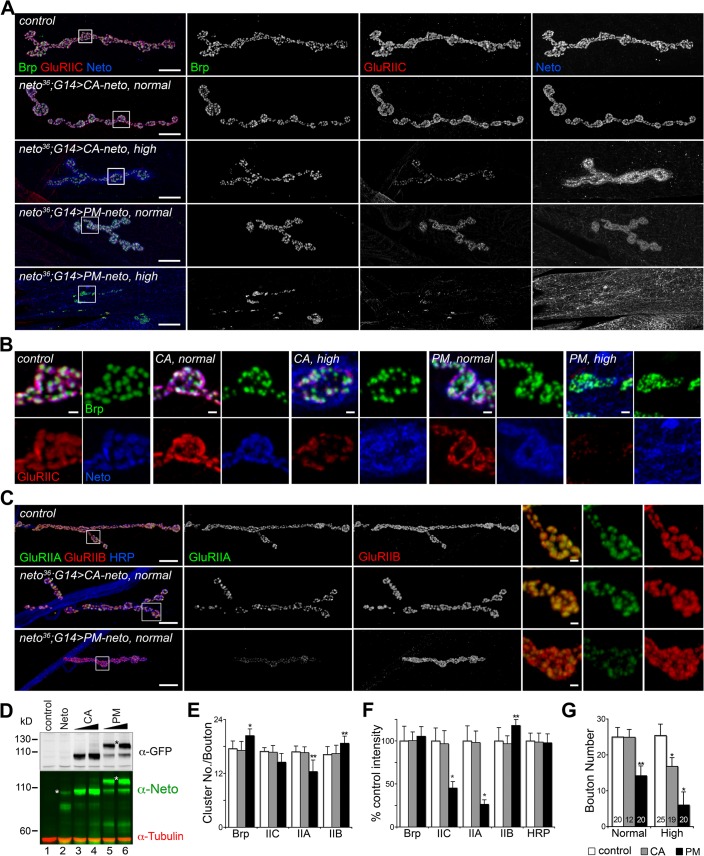
Prodomain processing affects Neto-mediated iGluR clustering. (A-B) Confocal images of NMJ4 (A) and bouton details (B) in larvae of indicated genotypes labeled for Brp (green), GluRIIC (red), and Neto (blue). Similar to *neto* transgenes, *CA-neto* induced dose-dependent gain-of-function NMJ phenotypes. In contrast, *PM-neto* transgenes severely disrupted the NMJ morphology and the synaptic contacts. (C) Confocal images of NMJ4 labeled for GluRIIA (green), GluRIIB (red), and HRP (blue) uncovered a drastic reduction of GluRIIA synaptic signals at PM-Neto rescued NMJs. (D) Western blot comparison of Neto expression levels in muscle extracts from third instar larvae rescued with: (i) untagged Neto (lane 2, *neto^36^;G14>neto-A3*); (ii) CA-Neto-GFP, normal (lane 3) and high (lane 4); and (iii) PM-Neto-GFP, normal (lane 5) and high (lane 6). (*) uncleaved proteins. (E-F) Quantification of various synaptic signals at *neto* null NMJs rescued with normal levels of CA- and PM-Neto. (G) Bouton numbers were severely reduced at PM-Neto rescued NMJs. The numbers of NMJs examined are indicated in each bar. Genotypes: CA-Neto normal (*neto^36^;G14>CA-neto-GFP-N4* at 18°C); CA-Neto high (*neto^36^;G14>CA-neto-GFP-N4* at 25°C); PM-Neto normal (*neto^36^;G14>PM-neto-GFP-D2* at 25°C); PM-Neto high (*neto^36^;G14>PM-neto-GFP-D1* at 25°C). Error bars indicate SEM. *; *p*<0.001, **; *p*<0.01. Bars: 10 μm, 1 μm in details.

Similar to the wild-type *neto* transgenes, moderate levels of CA-Neto rescued the NMJ morphology and iGluRs clustering defects of *neto* null mutants, while excess CA-Neto generated smaller NMJs with reduced iGluRs synaptic signals (Fig. 7A, B). In contrast, PM-Neto rescued NMJs developed abnormally irrespective of the expression levels. At moderate levels, PM-Neto distributed diffusely and disrupted the synaptic localization of iGluRs, in particular the type-A receptors (Fig. 7A-D, quantified in 7E, F). Animals rescued with high PM-Neto levels died during the early larval stages; the rare third instar escapers did not move and had severely altered NMJs with sparse boutons decorated by irregular Brp-positive aggregates and almost undetectable synaptic GluRIIC puncta (Fig. 7A, B, G). These data suggest that PM-Neto is inadequate for the proper recruitment and stabilization of iGluRs at postsynaptic locations even though PM-Neto appears to bind to GluRIIC in vivo and to enable embryos to hatch into larval stages (Fig. 6C, D). The severity of phenotypes at PM-Neto rescued synapses indicates that prodomain removal is required for iGluRs synaptic clustering, for development of postsynaptic structures, or both.

### Postsynaptic differentiation requires active Neto

**Fig 8:**
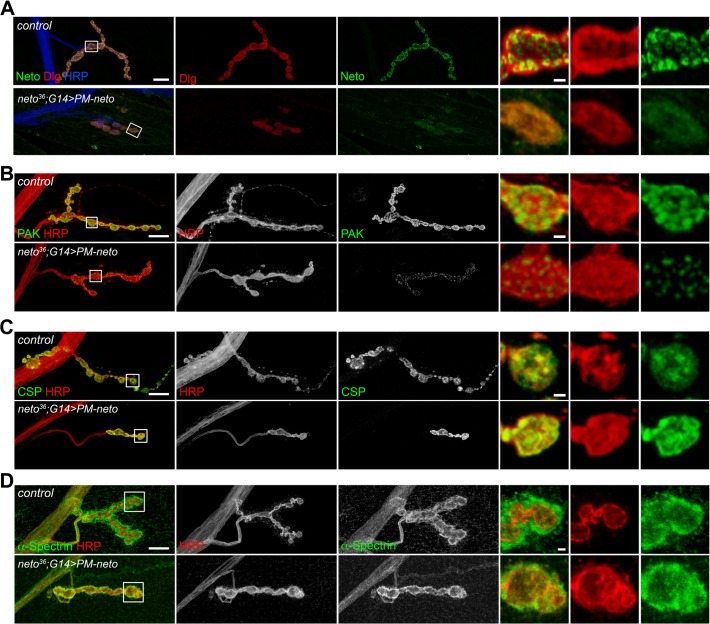
Postsynaptic differentiation requires processing of Neto prodomain. (A) Confocal images of NMJ4 (segment A4) in control and PM-Neto rescued (*G14>PM-neto-GFP-D2*) third instar larvae labeled for Neto (green), Dlg (red), and HRP (blue). The Neto and Dlg synaptic signals were diminished in intensity and appeared diffuse at PM-Neto NMJs. (B-D) Confocal images of NMJ4 boutons labeled for PAK (B), CSP (C) or α-Spectrin (D) (in green) and HRP (in red) revealed abnormal distribution of postsynaptic but not presynaptic proteins at PM-Neto rescued NMJs (*neto^36^;G14>PM-neto-GFP-D2)*. Bars: 10 μm, 1 μm in details.

Perisynaptic Dlg signals flank but do not co-localize with PSD components [54]. At control NMJ, Dlg appeared to surround the Neto-positive puncta (Fig. 8A). The synaptic accumulation of Dlg was severely reduced at PM-Neto rescued NMJs, without any detectable change in the level of Dlg protein in larval muscle. These mutant NMJs were hardly recognizable since both Dlg and Neto synaptic signals were diminished and distributed diffusely among very few boutons. Similar to iGluRs and Dlg, PAK did not accumulate at PM-Neto rescued NMJs (Fig. 8B). In contrast, the assembly of presynaptic components was not affected in PM-Neto rescued synapses: Brp and CSP showed discrete synaptic distributions (Figs 7, 8C). The severe postsynaptic defects at PM-Neto rescued NMJs were not accompanied by cytoskeletal disruption as indicated by normal α-Spectrin distribution (Fig. 8D). Thus, postsynaptic differentiation and organization of PSD structures appear to be specifically affected by Neto processing.

**Fig 9:**
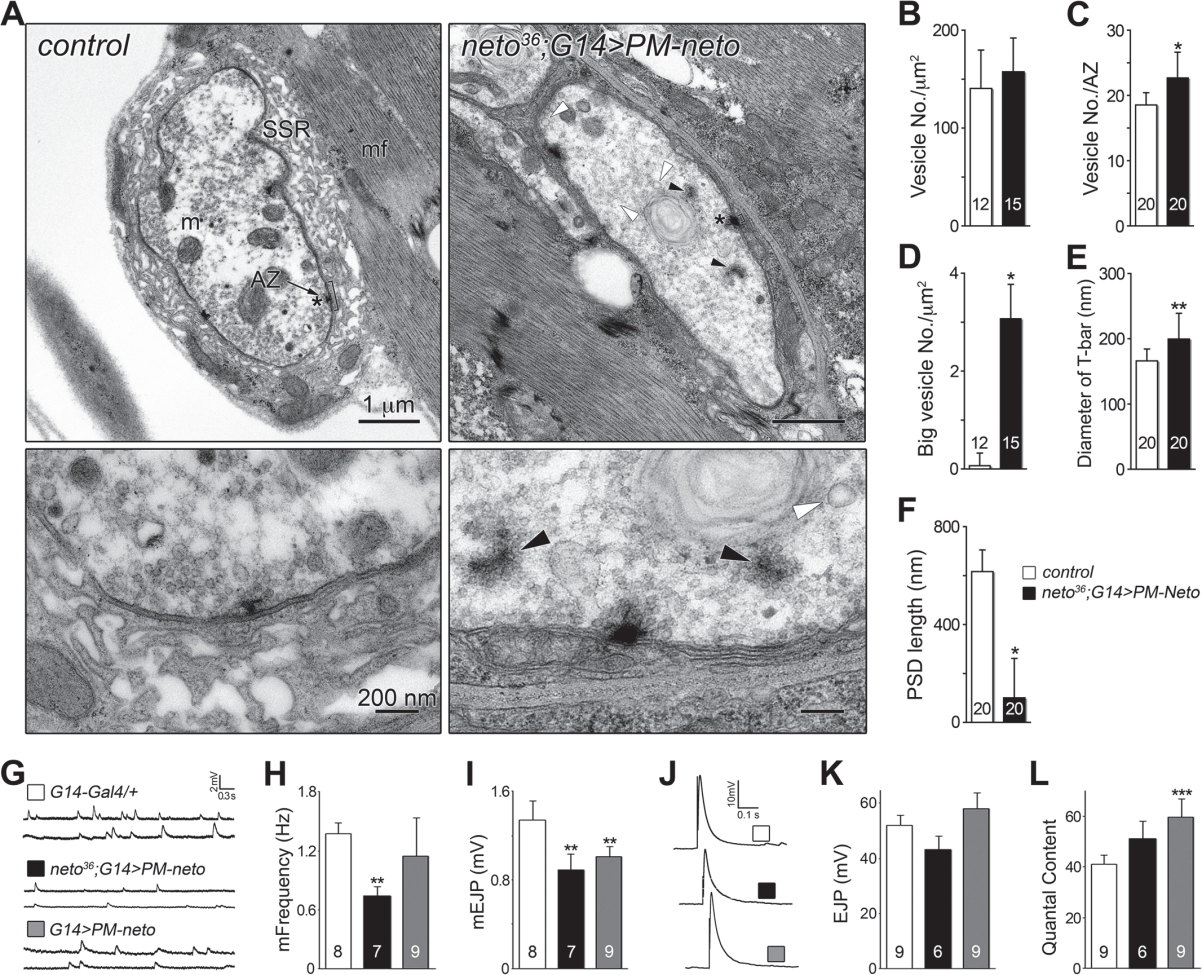
Neto prodomain affects synapse structure and function. (A) Electron micrographs of synaptic boutons from control and PM-Neto rescued (*G14>PM-neto-GFP-D2*) third instar larvae. Details are shown in lower panels and a larger field in S5 Fig.. The SSR, active zone (AZ), and PSDs (brackets) are indicated. SSR was densely packed around control type Ib boutons, but was severely disrupted at PM-Neto rescued boutons, which also showed loss of PSDs, floating T-bars (dark arrowheads), and enlarged synaptic vesicles (white arrowheads). (B-F) The numbers of synaptic vesicles, T-bar sizes, and PSDs length were compared. The numbers of boutons (B and D) or active zones (C, E and F) examined are indicated in each bar. (G-L) Electrophysiological recordings at PM-Neto rescued or overexpressed NMJ. Representative traces of spontaneous (G) and evoked (J) neurotransmitter release recorded from muscle 6 of indicated genotypes. The frequency and amplitude of mini EJPs were decreased in PM-Neto rescued NMJ (H-I). Evoked EJPs were largely normal (corrected values shown in K) due to presynaptic compensation (L). The numbers of NMJs examined are indicated in each bar. Error bars indicate SEM. *; *p*<0.001, **; *p*<0.01, ***; *p*<0.05.

The aberrant postsynaptic differentiation at PM-Neto rescued NMJs was also captured by electron micrographs of larval NMJs. These NMJs had rare boutons with no postsynaptic electron dense structures and no detectable SSR, and surrounded instead by dense ribosome fields or myofibrils (Figs 9A, B-F, and S5). The T-bar structures were often misshaped, collapsed or floating at PM-Neto boutons, suggesting that lack of Neto/iGluRs clustering affects proper assembly and organization of presynaptic structures. Larger T-Bars and synaptic vesicles at PM-Neto-rescued NMJs may reflect a homeostatic compensatory response to reduced postsynaptic receptors. Similar structures were reported in mutants with enhanced presynaptic release [10].

Physiological recordings indicated that the mini frequency was severely reduced at PM-Neto rescued NMJs consistent with drastically reduced synaptic iGluRs (Fig. 9G, H). The mini amplitude was also decreased, likely due to the preferential loss of type-A receptors at these synapses (Figs 7D, 9I). Consistent with the large vesicle seen in electron micrographs we occasionally observed very large minis at PM-Neto rescued NMJs. However, the evoked potentials were normal suggesting a presynaptic compensatory response (Fig. 9J-L). Thus, Neto processing is required for the normal density of postsynaptic iGluRs, but is not essential for triggering a compensatory increase in presynaptic release.

PM-Neto not only failed to cluster and stabilize the iGluRs at postsynaptic locations but it was also unable to support the recruitment of postsynaptic components, formation of PSDs, and stabilization of postsynaptic structures. The postsynaptic differentiation program was simply not initiated at PM-Neto rescued NMJs. Our data are consistent with a model in which Fur1-dependent processing activates Neto and allows it to function to stabilize iGluR complexes at synaptic sites. The prodomain may prevent the formation and/or maintenance of stable Neto/iGluR synaptic aggregates by obstructing Neto-mediated protein interactions. Lack of iGluRs clustering precludes the initiation of postsynaptic differentiation.

## Discussion

Clustering of *Drosophila* iGluRs at synaptic locations is limited by the essential subunits GluRIIC-E and by the obligatory auxiliary protein Neto [12–14,23]. Reduced Neto levels limit the iGluRs synaptic distribution and formation of postsynaptic specializations, without any change in the net levels of iGluRs or synaptic components [23,33]; excess Neto triggers a selective reduction of the synaptic iGluRs, but not other postsynaptic components, likely by sequestering iGluRs at extrajunctional locations. Neto activities are modulated by Furin-type proteases, which remove the inhibitory prodomain of Neto and enable Neto/iGluRs synaptic clustering and postsynaptic differentiation. Since Furins also process and activate multiple other substrates, including TGF-β ligands and extracellular matrix components, modulation of Neto activities by prodomain processing may serve as a way to coordinate synapse assembly with NMJ growth and development.

### Optimal Neto levels are required for NMJ development

The increase as well as the decrease of Neto levels affects the NMJ development, albeit with different consequences. Neto-deprived NMJs have diminished postsynaptic specializations and long branches, spanning over large muscle areas, suggesting that lack of postsynaptic receptors maintains the motor neurons in a growing, exploratory state. By contrast, NMJs with excess Neto are short and have normal accumulation of postsynaptic components. In fact, PAK and Dlg signals are slightly elevated at NMJs with excess Neto compared with control (S2 Fig.). Early accumulation of synaptic Dlg may restrict expansion of these NMJs and produce hypo-innervation. Interestingly, overexpression of Neto in the wild-type background (*G14>neto-A1*) induced gain-of-function phenotypes slightly milder than when the same transgene replaced the endogenous *neto* in rescue experiments (compare the last two columns in Fig. 1A). This could be due to the different genetic backgrounds or may indicate additional Neto functions that are missing at *neto-A1*-rescued NMJs.

Physiological studies also captured the differences between postsynaptic iGluR receptor fields at different Neto levels. Neto-deprived NMJs in *neto* hypomorphs or RNAi experiments have severely reduced mini frequency consistent with their reduced postsynaptic iGluRs density (Fig. 1C-F and [23,33]). Strong reduction of postsynaptic Neto levels induced a reduction of EJP amplitudes, suggesting that Neto deprivation interferes with the normal homeostatic mechanisms. Similar to iGluRs-deprived synapses, lack of Neto may render these synapses “beyond repair” [12,14]. In contrast, the NMJ physiological parameters appeared more to be resilient to excess Neto since addition of moderate levels of Neto did not affect the mEJP and EJP amplitude. However, high levels of excess Neto (*G14>neto-A1*) induced a significant decrease of mEJP frequency, consistent with the reduced synaptic and increased extrasynaptic iGluRs observed at these NMJs (Figs 1, 2, 4).

At central glutamatergic synapses in vertebrates, synaptic receptors are cycling into and out of the synapses indicating that synapses behave as donors or acceptors for receptors, and the extrasynaptic receptors function as a reserve pool [2]. At the *Drosophila* NMJ, the iGluRs are recruited to the nascent synapses from extrajunctional receptor pools, but are stably integrated in synaptic aggregates with very low turnover [22]. In the absence of Neto, or any essential iGluR subunit, the iGluRs are not recruited at synaptic locations [55]. Conversely, excess Neto induces accumulation of iGluR-positive puncta at extrajunctional locations (Figs 2, 4). This is different than overexpression of any of the essential iGluR subunits, which don’t show gain-of-function phenotypes, presumably because other subunits are limiting [11]. Furthermore, the iGluR complexes appear to be on the muscle membrane at suboptimal Neto levels since they are accessible by antibodies in the absence of detergents (Fig. 4 and [23]). Likewise, Neto proteins from worms and mammals appear to have no roles (or very modest ones) in the surface delivery of the iGluRs [25,26]. We speculate that Neto binds iGluRs on the cell surface and engages in extracellular and/or intracellular interactions that enable the recruitment and clustering of iGluRs at synaptic sites. In this scenario, reduced Neto levels are inefficient for the iGluRs synaptic trafficking and clustering, whereas excess Neto may engage in protein interactions that sequester iGluRs at ectopic locations.

### Modulation of synaptic Neto activities

At the *Drosophila* NMJ, Neto activities are regulated by Fur1-dependent limited proteolysis. The removal of Neto prodomain appears to be essential for the stabilization of iGluRs at PSDs. Lack of iGluRs stabilization precludes postsynaptic differentiation although the receptors are functional (Figs 8, 9). Thus, synapse activity does not trigger iGluRs clustering or postsynaptic differentiation; instead, stabilization of iGluRs at synaptic sites initiates the recruitment of PSD components and assembly of postsynaptic structures.

It has been proposed that a neuron secreted molecule triggers clustering of iGluRs at *Drosophila* NMJ [18,20,56]. Secreted molecule(s) may mediate iGluRs clustering by binding and trapping Neto/iGluR complexes at new synapses. Mind the gap (Mtg) is a neuronal protein reported to organize the synaptic cleft [57]. In *mtg* null mutant embryos, Neto and iGluRs form aggregates comparable in size with control clusters, but which fail to concentrate at nascent synapses [55]. Unfortunately, we could not detect in vitro interactions between Neto and Mtg. But while the molecular nature of the “trapping” mechanism remains to be determined, our study demonstrates that this process requires the removal of Neto prodomain.

The Neto prodomain does not interfere with targeting and apical localization of Neto, nor does it affect its ability to bind iGluRs and form complexes, but it appears to preclude Neto engagement in protein interactions required for the formation of iGluR synaptic clusters. Neto CUB1 domain interacts with itself, but self-association is not enough to explain the formation of large iGluR aggregates. Prodomains could mediate binding to extracellular factors, such as heparan proteoglycans, fibrillin and perlecan that protect the active molecules and modulate their extracellular distribution [58,59]. Our study does not address a role for Neto prodomain in binding to extracellular molecules that modulate Neto distribution. The prodomains could also function as chaperones that allow proper folding of biologically active molecules, such as TGF-β-type ligands [34]. However, Neto prodomain is unlikely to play a role in the folding and secretion of Neto because CA-Neto is functional and induces NMJ gain-of-function phenotypes similar to excess Neto. Alternatively, the prodomain could maintain Neto in an inactive form, thus limiting clustering and stable incorporation of Neto/iGluR complexes at PSDs. Similar regulation has been described for the Tolloid/BMP-1 family of enzymes: their prodomains must be removed before the catalytic domains could assume active conformations [60]. It is tempting to speculate that the prodomain masks Neto extracellular domain(s) and prevents interactions required for iGluR clustering at PSDs.

**Fig 10:**
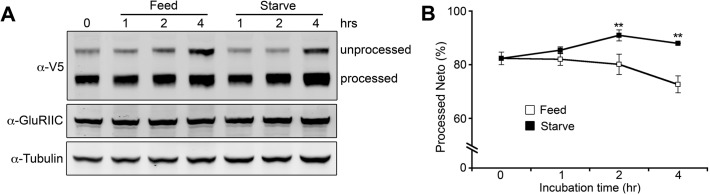
Modulation of Neto processing. (A) Western blot comparison of the extent of Neto processing in fed and starved larvae. Third instar larvae (*neto^36^;24B>neto-V5*) were separated into two groups: one group was fed with yeast paste and the other was starved. Muscle extracts were analyzed at indicated time points and the relative ratio of processed Neto was calculated against total Neto (B). Values were averaged from three individual experiments. **; *p*<0.01

Is Neto processing a general step in Neto passage through the secretory pathway or could it actively modulate Neto activity/ availability? To test if processing plays an active role in regulating Neto function we compared the changes in Neto processing in larvae with hunger-induced increase of locomotion [61]. The proportion of processed Neto increased in starved larvae and decreased in fed animals (Fig. 10), indicating that Neto processing indeed changes in response to an increase in locomotion and/or due to starvation. While this analysis cannot distinguish between the two possibilities, Neto processing emerges as an active mechanism to control the level of Neto available for effective iGluRs recruitment at PSDs.

Neto processing/activation phenomenon appears to be highly conserved in insects. Most insects have glutamatergic NMJs, and their genomes encode for Neto proteins with prodomains and Furin minimal sites (R-X-X-R) preceding the first CUB domain. For example, Neto proteins in *Apis florea* and *Apis mellifera* share an R-Q-M-R motif at positions equivalent to the Furin site in *Drosophila* Neto. In all cases, the Furin consensus sites are suboptimal suggesting that processing of insect Neto proteins will be slow and restricted by Furin activities. Furins cleave their substrates mainly in late Golgi, though recent data indicate that Furins also function at the cell surface and in the extracellular space [62]. Interestingly, Fur1 also cleaves and activates TGF-β-type ligands, including Gbb, Maverick and Dawdle, which are secreted from muscle and glia and control NMJ development [37,39,40]. This raises the possibility that Fur1 synchronizes the activation of Neto and TGF-β factors and may serve as a means to coordinate synapse assembly with NMJ growth.

This study does not exclude other mechanisms that may regulate the density of synaptic iGluR, such as local insertion of iGluRs from intracellular vesicles [63]. Nonetheless, our study demonstrates that Neto activation by prodomain processing plays an important role in the regulation of iGluR trafficking and clustering at synapses. Trafficking of Neto itself or Neto/iGluR complexes on the muscle membrane may be further controlled by cellular signals that modulate the intracellular domain of Neto and regulate its coupling with scaffold and motor complexes. In fact, *Drosophila neto* locus codes for two isoforms generated by alternative splicing that differ in their intracellular domains. Both intracellular domains contain multiple putative phosphorylation sites, raising the possibility of rich modulation of Neto/iGluRs distribution in the striated muscle.

## Materials and Methods

### Fly stocks

Fly lines were generated by standard germline transformation of pUAST-based plasmids containing various *neto* constructs (BestGene, Inc). Other stocks used in this study were as follows: *neto* null and hypomorph alleles, *neto* ^*36*^ and respectively *neto* ^*109*^ [23], *neto* ^*RNAi*^ [33], *G14-Gal4* and *MHC-Gal4* (obtained from C. Goodman, University of California at Berkeley), *da-Gal4* (BL-5460), *24B-Gal4* (BL-1716), and *elav-Gal4* (BL-8760). For *RNAi*-mediated knockout we used the following TRiP lines generated by the Transgenic RNAi Project: *GluRIIC* (*P[TRiP*.*JF01854}attP2*), and *fur1 (P[TRiP*.*GL01340] attP40)*. The control is *y* ^*1*^ *w* ^*1118*^ unless otherwise specified.

For rescue analyses, *neto* transgenes were introduced into *neto* ^*36*^ null mutant background using tissue-specific promoters. Since *neto* is on the X-chromosome we used only *FM7-GFP* balanced stocks to eliminate any meiotic non-disjunction event. The F1 progenies were genotyped during late embryogenesis and reared at the indicated temperatures. After 24 hours, crawling larvae were removed, counted, and kept at the same temperatures for further analyses or adult viability testing.

### Molecular constructs

Neto variants were generated using QuikChange site-directed mutagenesis kit (Stratagene) as described previously [23]. CA-Neto has a deletion that joins A^51^-Q^130^ and loops out the Neto prodomain. PM-Neto has two point mutations: R^123^I and R^126^I. Double-tagged Neto constructs were generated by QuikChange loop-in of various Neto fragments in a previously described AcPA-SP-Myc-V5/His plasmid [64]. This actin promoter/terminator plasmid contains the sequences coding for the Tolloid-related signal peptide, the 5xMyc cassette, a multiple cloning site, followed by the V5 and RGS-6xHis epitopes. All constructs were verified by DNA sequencing.

For RNA interference, PCR primers for Furins that carry the T7 promoter sequence at the 5’ end were designed as previously described [36]. The primers were as follows:

dFur1-F 5’-TAATACGACTCACTATAGGGACGCAAAGATCCTCTGTGGCA;

dFur1-R 5’- TAATACGACTCACTATAGGGACATTGCTCCCGGAACTGC;

dFur2-F 5’- TAATACGACTCACTATAGGGACGCTAGAGGCCAATCCGGAA;

dFur2-R 5’- TAATACGACTCACTATAGGGACCCTTCTCGCCCCAAAAGTG;

Amon-F 5’- TAATACGACTCACTATAGGGACCCACATGGAGCTGGCTGT;

Amon-R 5’- TAATACGACTCACTATAGGGACCCTGACTTTGCCGCCATT.

PCR products were amplified from genomic DNA or S2 cells cDNA. In vitro transcribed dsRNA was produced using the MEGAscript kit (Ambion). RNAi treatment was carried out by transfections of 5 mg/ml of dsRNA into S2 cells.

### RT-PCR

S2 cells were transfected with indicated constructs and harvested after five days incubation. Total RNA was extracted using TRIZOL reagent (Invitrogen) according to manufacturer's instructions. AccuScript High Fidelity First-Strand cDNA Synthesis Kit (Agilent) was used to generate cDNAs from the extracted total RNAs according to manufacturer’s instructions. PCR reaction for each target gene was executed using the cDNAs as templates with specific primer pairs (above) and β-Actin as a reaction standard (Actin-Forward: 5’-CTGGCACCACACCTTCTACAATG-3’, Actin-Reverse: 5’-GCTTCTCCTTGATGTCACGGAC-3’).

### Immunohistology

Wandering third instar larvae were dissected as described previously in ice-cooled Ca^2+^-free HL-3 solution [65,66]. Dissecting larval tissues were fixed in either 4% formaldehyde or Bouin's fixative (Polysciences, Inc.) for 20 min or 5 min respectively. PBS containing 0.5% Triton X-100 was used for washing and antibody reaction. For detergent-free staining, 1X PBS was used. Primary antibodies from Developmental Studies Hybridoma Bank were used at the following dilutions: mouse anti-GluRIIA (MH2B), 1:100; mouse anti-Dlg (4F3), 1:1000; mouse anti-Brp (Nc82), 1:100; mouse anti-CSP (6D6), 1:100; mouse anti-α-spectrin (3A9), 1:100. Other primary antibodies were as follows: rat anti-Neto, 1:1000 [23], rabbit anti-GluRIIB, 1:2000 (a gift from David Featherstone) [67]; rabbit anti-GluRIIC, 1:2000 [33]; rabbit anti-PAK, 1:2000 (a gift from Nicholas Harden) [68]; FITC-, rhodamine-, and Cy5-conjugated goat anti-HRP, 1:1000 (Jackson ImmunoResearch Laboratories, Inc.). Alexa Fluor 488-, Alexa Fluor 568-, and Alexa Fluor 647-conjugated secondary antibodies (Molecular Probes) were used at 1:400. All samples were mounted with ProLong Gold reagent (Invitrogen) and incubated for 24 hours at RT. Confocal images were acquired using Carl Zeiss LSM 780 or 510 laser scanning microscopic system with Plan-Apochromat 63X/1.4 oil DIC objective using ZEN software. Z-stacked images were collected, processed, and analyzed using Imaris X64 (7.6.0, Bitplane) or ImageJ (NIH) software. In each experiment, samples of different genotypes were processed simultaneously and imaged under identical confocal settings. To quantify fluorescence intensities, confocal regions of interest (ROIs) surrounding anti-HRP immunoreactivities were selected and the signals measured individually at NMJs from ten or more different larvae for each genotype (number of samples is indicated in the graph bar). The signal intensities were calculated relative to HRP volume and subsequently normalized to control. For the extrajunctional, cell surface GluRIIC staining, where the GluRIIC positive signals are predominantly in the form of puncta at both Neto-depleted and Neto-excess NMJs, intensities from several size-matched areas of the muscles were collected and averaged using Image J software. The numbers of muscles analyzed per genotype are indicated inside the bars. Quantification of NMJ morphological features was performed at muscle 4 of abdominal segment 4 using the filament tracing function of Imaris software. Boutons were counted manually, while blind to the genotype, using anti-HRP and anti-Dlg staining. Statistical analyses were performed using the Student’s t-test with a two-tailed distribution and a two-sample unequal variance. All graphs represent mean value of all samples of the given genotype ± SEM.

### Protein production and analysis

Transiently transfected *Drosophila* S2 cells were used for producing recombinant proteins as previously described [69]. The S2 cells were maintained in M3 (Shields and Sang M3 insect medium, Sigma) with 1x insect medium supplement (Sigma) and Penicillin/Streptomycin (Sigma), and sub-cultured every 7 days at 2 X 10^6^ cells/ml. For the transfection, dimethyldioctadecyl-ammonium bromide (DDAB) solution (250 μg/ml) was mixed with M3 media at 1:2 ratio and incubated 5 min at RT, then the DNA was added to the DDAB-M3 mixture (1μg of plasmid DNA to 100 μl suspension). The mixture was incubated for 20 min and transfected into S2 cells (100 μl mixture to 2 X 10^6^ cells/ml culture). After five days, the secreted proteins were collected for analysis, and membrane proteins were extracted by homogenizing cells in lysis buffer (50 mM Hepes-NaOH, 150 mM NaCl, 0.2 mM EDTA, 0.5% NP-40, 0.1% SDS, 2mM AEBSF [MP BIO], and protease inhibitor cocktail [Roche]) for 30 min on ice. The lysates were collected by centrifugation at 13,000rpm for 30 min at 4°C, separated by SDS-PAGE on 4%–12% NuPAGE gels (Invitrogen) and transferred onto PVDF membranes (Millipore). Primary antibodies were used at the following dilutions: rat anti-Neto, 1:1000; chicken anti-GFP (Abcam), 1:2000; anti-GluRIIC, 1:1000; anti-tubulin (Sigma), 1:1000. Immune complexes were visualized using secondary antibodies coupled with IR-Dye 700 or IR-Dye 800 followed by scanning with the Odyssey infrared imaging system (Li-Cor Biosciences).

To analyze muscle proteins, wandering third instar larvae were dissected, and the body walls were mechanically homogenized in lysis buffer for 30 min on ice. The lysates were analyzed by Western blotting. For co-immunoprecipitation, the lysates were incubated with rabbit anti-GFP antibody (Invitrogen) for 1 hr at 4°C. Protein A/G UltraLink Resin (50% slurry, Thermo Scientific) was added and incubated overnight at 4°C. The beads were washed with lysis buffer. Proteins were eluted with 1x SDS sample buffer and analyzed by Western blotting.

Secreted and processed Neto fragment (CUB1-V5/His) was purified using His-Trap affinity column equipped with AKTA FPLC system (Pharmacia) and separated by SDS-PAGE. A specific gel band was isolated and analyzed at Microchemistry and Proteomics Analysis Facility, Harvard University.

### Electron microscopy

Wandering third instar larvae were dissected in Jan's saline containing 0.1 mM Ca^2+^ and processed as previously described [70]. Dissected larvae were fixed in EM fixative (4% *p*-formaldehyde, 1% glutaraldehyde, 0.1 M sodium cacodylate, and 2 mM MgCl_2_, pH 7.2) for 20 min at room temperature followed by incubation overnight at 4°C, then washed extensively (0.1 M sodium cacodylate, and 132 mM sucrose, pH 7.2). The samples were processed and analyzed at the Microscopy and Imaging Core Facility, NICHD.

### Electrophysiology

The standard larval body wall muscle preparation first developed by Jan and Jan (1976) was used for electrophysiological recordings [71,72]. Wandering third instar larvae were dissected in physiological saline HL-3 [65], washed, and immersed in HL-3 containing 0.8 mM Ca^2+^ using a custom microscope stage system [73]. The nerve roots were cut near the exiting site of the ventral nerve cord so that a suction electrode could pick up the motor nerve later. Intracellular recordings were made from muscle 6. Data were used when the input resistance of the muscle was >5 MΩ and the resting membrane potential was between −60 mV and −80 mV for the entire duration of the experiment. The input resistance of the recording microelectrode (backfilled with 3 M KCl) ranged from 20 to 25 MΩ. Muscle synaptic potentials were recorded using Axon Clamp 2B amplifier (Axon Instruments) and pClamp software. Following motor nerve stimulation with a suction electrode (100 μsec, 5 V), evoked EJPs were recorded. Three to five EJPs evoked by low frequency of stimulation (0.1 Hz) were averaged. For mini recordings, TTX (1 μM) was added to prevent evoked release [65]. To calculate mEJP mean amplitudes, 50–100 events from each muscle were measured and averaged using the Mini Analysis program (Synaptosoft). Minis with a slow rise and falling time arising from neighboring electrically coupled muscle cells were excluded from analysis [72,74]. In addition, when comparing mini sizes between preparations, the Kolmogorov-Smirnov test was administrated. Quantal content was calculated by dividing the mean EJP by the mean mEJP after correction of EJP amplitude for nonlinear summation according to the methods described [75,76]. Corrected EJP amplitude = E[Ln[E/(E − recorded EJP)]], where E is the difference between reversal potential and resting potential. The reversal potential used in this correction was 0 mV [75,77]. Data are presented as mean ± SEM, unless otherwise specified; EJP amplitudes and quantal contents after the nonlinear correction are shown. A one-way analysis of variance followed by Tukey's HSD test was used to assess statistically significant differences among the genotypes. Differences were considered significant at *p < 0*.*05*.

## Supporting Information

S1 Fig Quantification of Neto levels and their ability to rescue *neto* null mutants. (A) Confocal images of third instar NMJ controls as indicated (muscle 4, A4) immunostained against Neto (green), and HRP (red). Bars: 10 μm. (B-C) The ability of various transgenes to rescue *neto* null mutants was measured as percentage of embryos hatching into larval stages (B) and adults (C). (D-E) The length and number of branch points of NMJs at various Neto levels, as illustrated in Fig. 1A. Neto-deprived NMJs were spread out while excess Neto induced formation of shorter NMJs. The numbers of NMJs examined are indicated in each bar.
(TIF)

S2 Fig Excess Neto does not perturb the distribution of various synaptic components. (A-D) Confocal images of third instar NMJs (muscle 4, A4) and bouton details labeled for HRP (in red) and PAK (A), Dlg (B), α-spectrin (C), or CSP (D) (in green). (E-F) Quantification of the number of synaptic clusters (E), and relative intensity (F) are indicated. Error bars indicate SEM. *; *p*<0.001, ***; *p*<0.05. Bars: 10 μm, 1 μm in details.
(TIF)

S3 Fig Tagged Neto variants recapitulate the gain-of-function phenotypes observed at NMJ with excess untagged Neto. (A) Confocal images of third instar NMJ (muscle 4, A4) immunostained against Dlg (green), and HRP (red). Compared with the control, and similar to NMJ with excess Neto, the *neto* null NMJs rescued with tagged *neto* transgenes (*neto^36^;24B>neto-V5 or neto^36^;G14>neto-GFP*) exhibited normal Dlg synaptic signals (B) but reduced NMJ size (C). (D-G) Muscle expression of Neto-GFP rescued the neurotransmission in *neto* mutants to control levels (*G14-Gal4/+)*. The numbers of NMJs examined are indicated in each bar. Error bars indicate SEM. *; *p*<0.001. Bars: 10 μm, 1 μm in details.
(TIF)

S4 Fig Fur1 is required both pre- and postsynaptically at the *Drosophila* NMJ. (A-B) Confocal images of third instar NMJ (muscle 4, A4) immunostained against Brp (green) and GluRIIC (red) (A); or GluRIIA (green) and pMad (red) (B) and Neto (blue). Knockdown of Fur1 in either muscles (*G14>fur1* ^*RNAi*^ and *24B>fur1* ^*RNAi*^) or neurons (*elav>fur1* ^*RNAi*^) induced mild reduction in the number of synaptic clusters but drastic decrease in the intensity of all synaptic signals examined (quantified in C-D). Knockdown of Fur1 also produced significant reduction of NMJ size (E). The numbers of NMJs examined were indicated in each bar. Error bars indicate SEM. *; *p*<0.001, **; *p*<0.01, ***; *p*<0.05. Bars: 10 μm.
(TIF)

S5 Fig Ultrastructural defects at PM-Neto-rescued NMJs. Large field from an electron micrograph of synaptic boutons from PM-Neto rescued (*G14>PM-neto-GFP-D2*) third instar larvae.
(TIF)
