# Supplementary figures and images for: Prodomain Removal Enables Neto to Stabilize Glutamate Receptors at the Drosophila Neuromuscular Junction
Source: PLoS Genet. 2015 Feb 27;11(2):e1004988. doi: 10.1371/journal.pgen.1004988 (PMC4344203; doi:10.1371/journal.pgen.1004988)

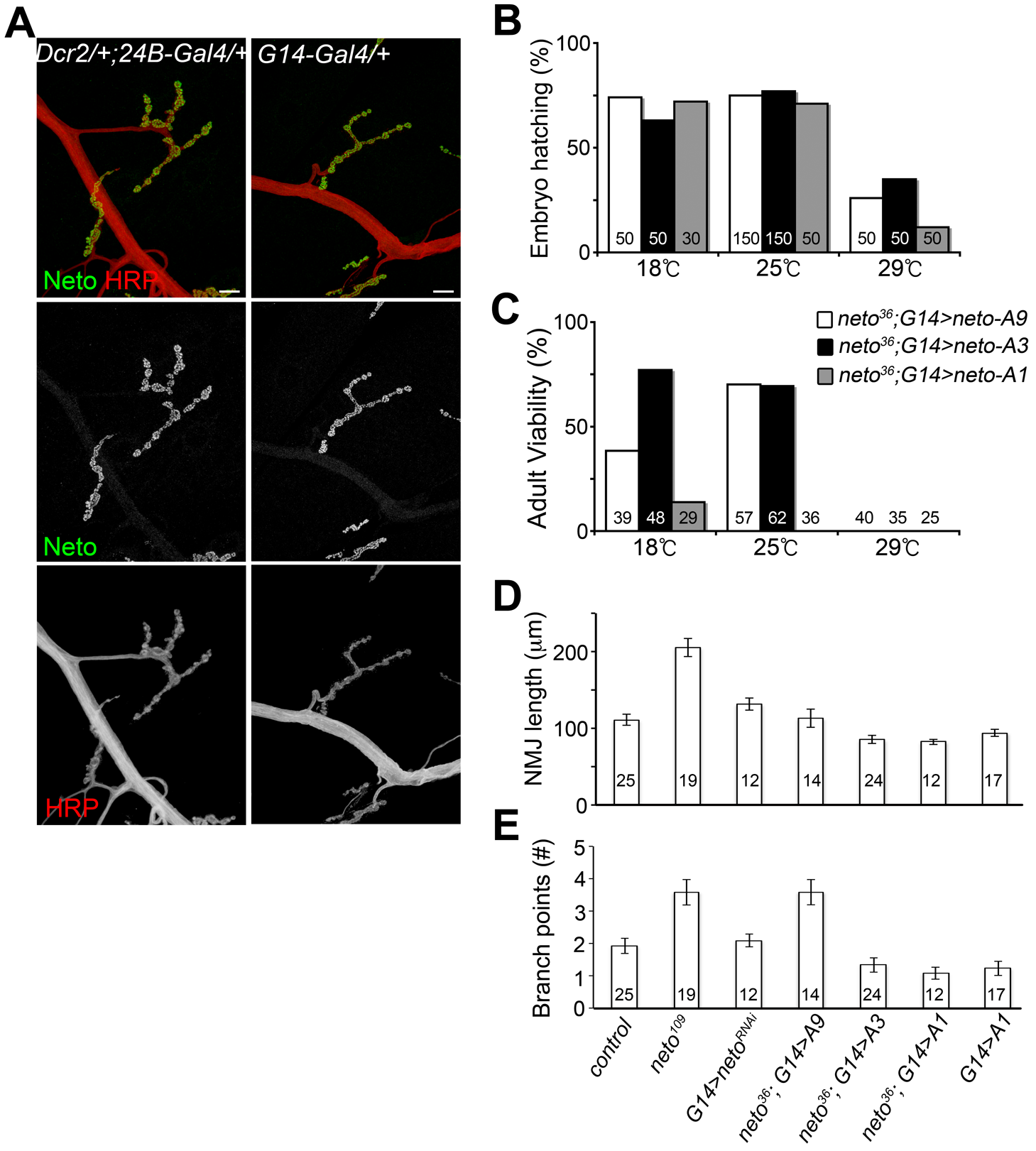

Supplement: S1 Fig — (A) Confocal images of third instar NMJ controls as indicated (muscle 4, A4) immunostained against Neto (green), and HRP (red). Bars: 10 μm. (B-C) The ability of various transgenes to rescue neto null mutants was measured as percentage of embryos hatching into larval stages (B) and adults (C). (D-E) The length and number of branch points of NMJs at various Neto levels, as illustrated in Fig. 1A. Neto-deprived NMJs were spread out while excess Neto induced formation of shorter NMJs. The numbers of NMJs examined are indicated in each bar. (TIF) [file pgen.1004988.s001.tif]

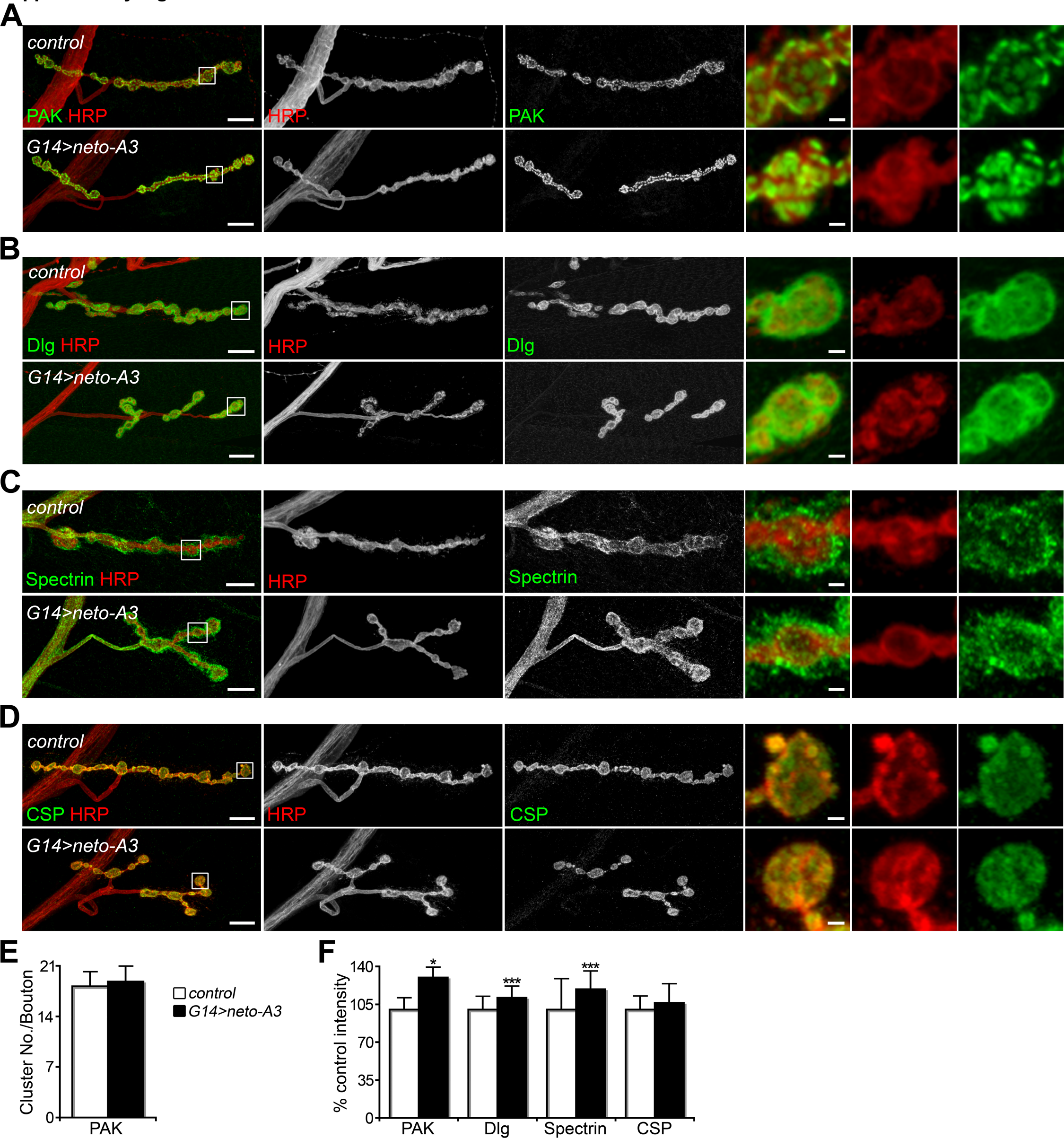

Supplement: S2 Fig — (A-D) Confocal images of third instar NMJs (muscle 4, A4) and bouton details labeled for HRP (in red) and PAK (A), Dlg (B), α-spectrin (C), or CSP (D) (in green). (E-F) Quantification of the number of synaptic clusters (E), and relative intensity (F) are indicated. Error bars indicate SEM. *; p<0.001, ***; p<0.05. Bars: 10 μm, 1 μm in details. (TIF) [file pgen.1004988.s002.tif]

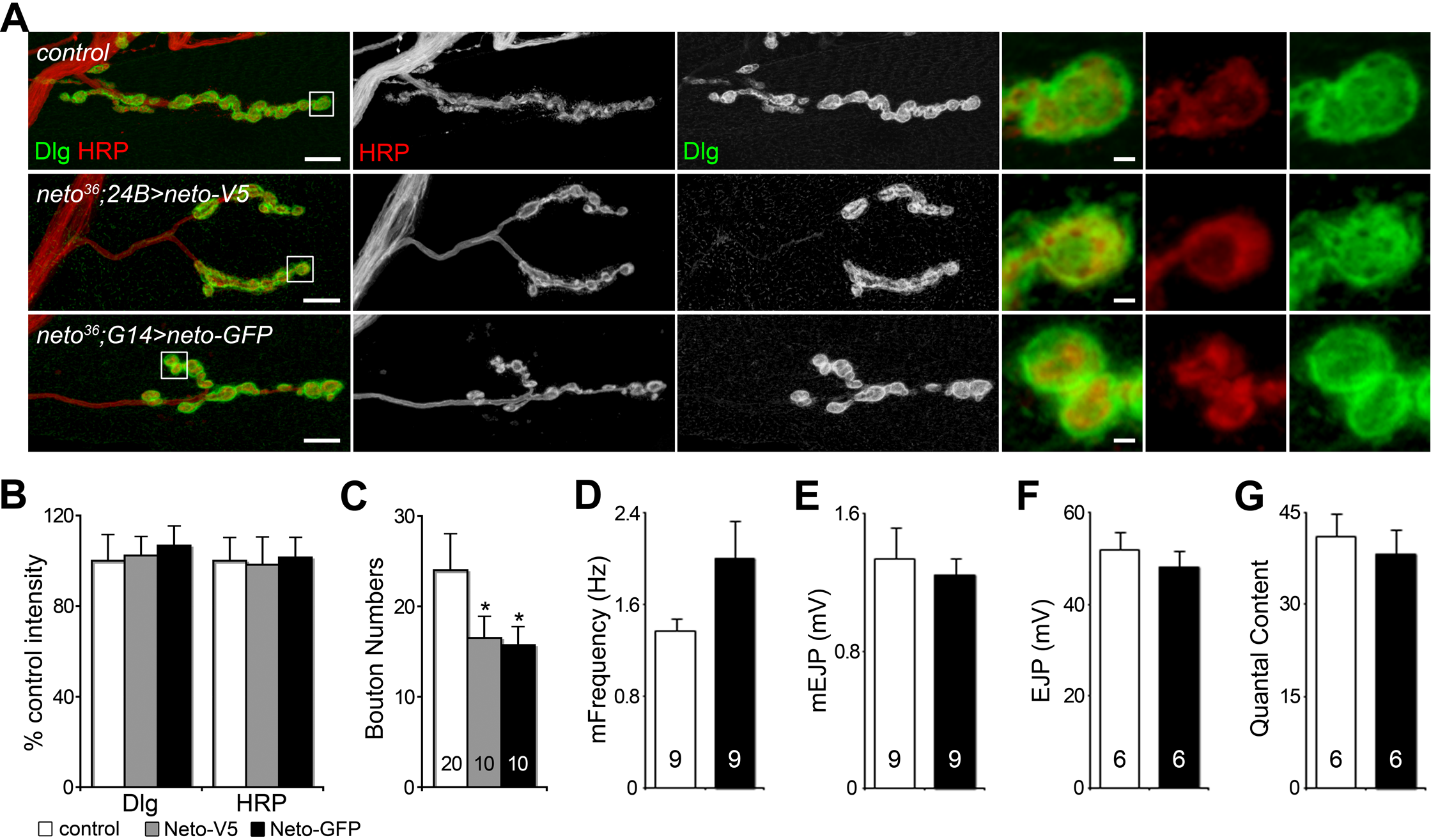

Supplement: S3 Fig — (A) Confocal images of third instar NMJ (muscle 4, A4) immunostained against Dlg (green), and HRP (red). Compared with the control, and similar to NMJ with excess Neto, the neto null NMJs rescued with tagged neto transgenes (neto36;24B>neto-V5 or neto36;G14>neto-GFP) exhibited normal Dlg synaptic signals (B) but reduced NMJ size (C). (D-G) Muscle expression of Neto-GFP rescued the neurotransmission in neto mutants to control levels (G14-Gal4/+). The numbers of NMJs examined are indicated in each bar. Error bars indicate SEM. *; p<0.001. Bars: 10 μm, 1 μm in details. (TIF) [file pgen.1004988.s003.tif]

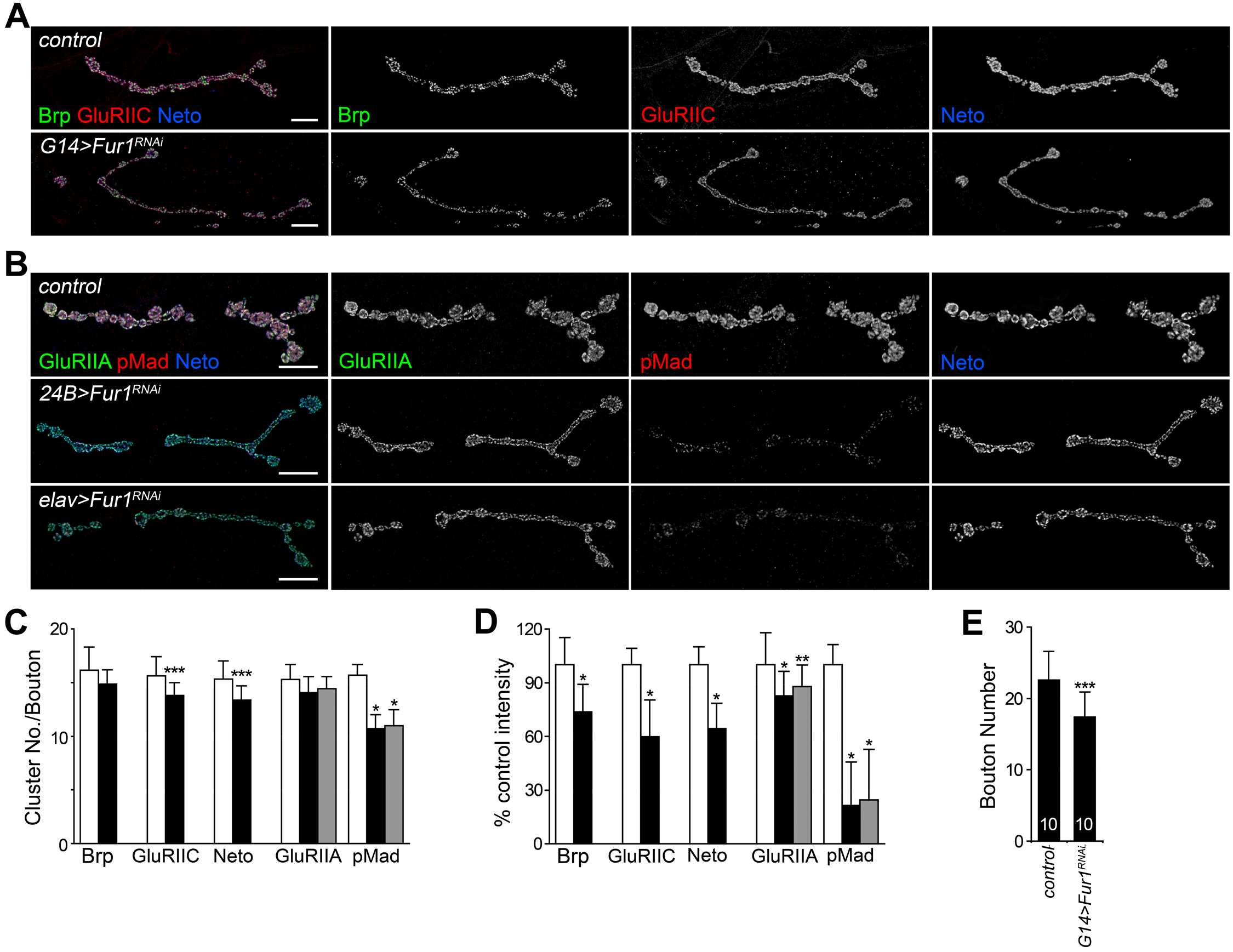

Supplement: S4 Fig — (A-B) Confocal images of third instar NMJ (muscle 4, A4) immunostained against Brp (green) and GluRIIC (red) (A); or GluRIIA (green) and pMad (red) (B) and Neto (blue). Knockdown of Fur1 in either muscles (G14>fur1 RNAi and 24B>fur1 RNAi) or neurons (elav>fur1 RNAi) induced mild reduction in the number of synaptic clusters but drastic decrease in the intensity of all synaptic signals examined (quantified in C-D). Knockdown of Fur1 also produced significant reduction of NMJ size (E). The numbers of NMJs examined were indicated in each bar. Error bars indicate SEM. *; p<0.001, **; p<0.01, ***; p<0.05. Bars: 10 μm. (TIF) [file pgen.1004988.s004.tif]

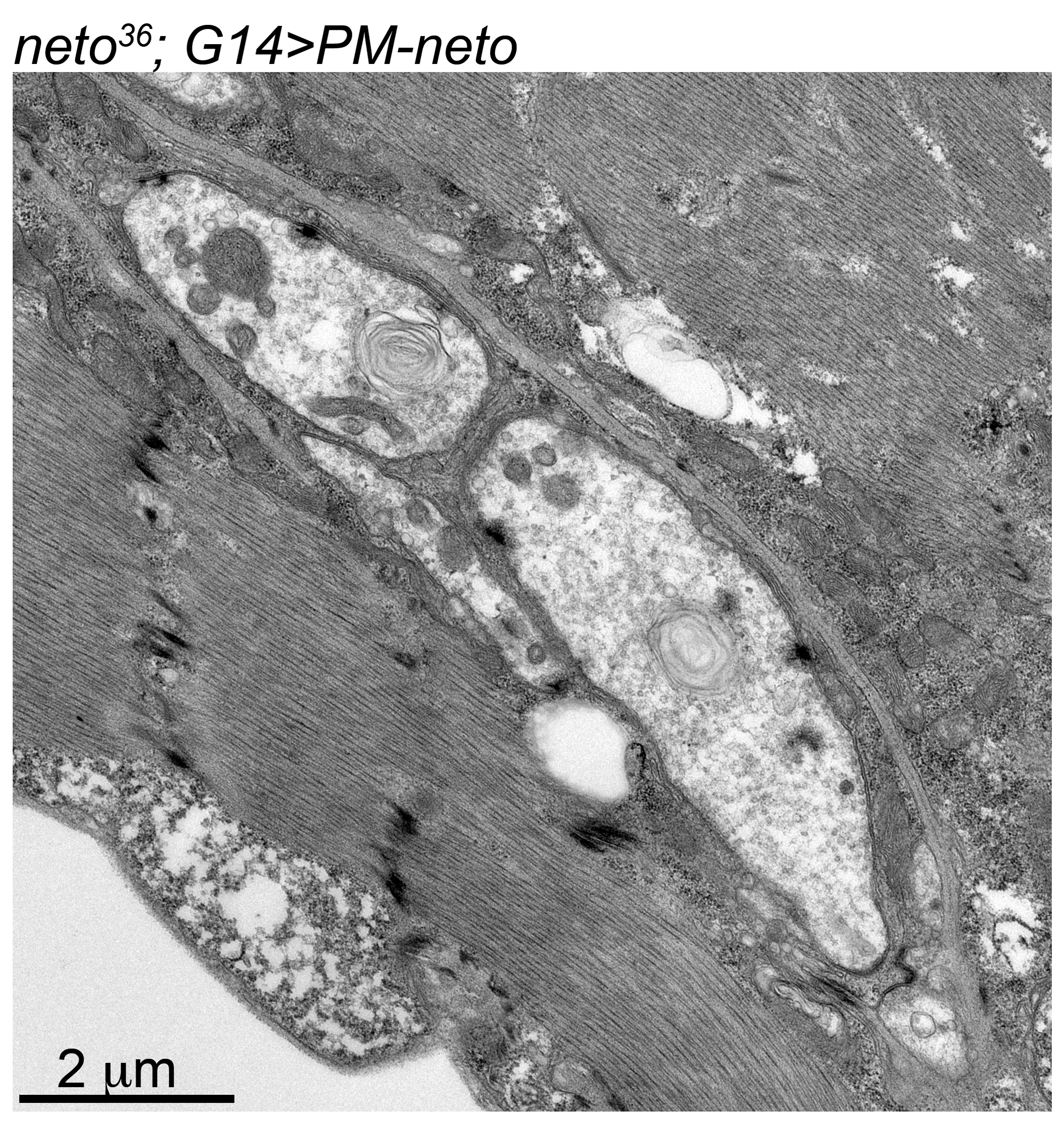

Supplement: S5 Fig — Large field from an electron micrograph of synaptic boutons from PM-Neto rescued (G14>PM-neto-GFP-D2) third instar larvae. (TIF) [file pgen.1004988.s005.tif]
